# Serum neutrophil gelatinase-associated lipocalin as a potential biomarker for cognitive decline in spinal cord injury

**DOI:** 10.3389/fneur.2023.1120446

**Published:** 2023-03-06

**Authors:** Qinghao Zhang, Ziteng Li, Liangyu Xie, Shengnan Cao, Zhonghao Cui, Bin Shi, Yuanzhen Chen

**Affiliations:** ^1^Bone Biomechanics Engineering Laboratory of Shandong Province, Shandong Medicinal Biotechnology Center (School of Biomedical Sciences), Neck-Shoulder and Lumbocrural Pain Hospital of Shandong First Medical University, Shandong First Medical University & Shandong Academy of Medical Sciences, Jinan, Shandong, China; ^2^School of Acupuncture and Tuina, Shandong University of Traditional Chinese Medicine, Jinan, China

**Keywords:** neutrophil gelatinase-associated lipocalin, biomarker, cognitive, spinal cord injury, serum

## Abstract

**Objective:**

Neutrophil gelatinase-associated lipoprotein (NGAL), a protein encoded by the lipocalcin-2 (LCN2) gene, has been reported to be involved in multiple processes of innate immunity, but its relationship with spinal cord injury (SCI) remains unclear. This study set out to determine whether NGAL played a role in the development of cognitive impairment following SCI.

**Methods:**

At the Neck-Shoulder and Lumbocrural Pain Hospital, a total of 100 SCI patients and 72 controls were enrolled in the study through recruitment. Through questionnaires, baseline data on the participants' age, gender, education level, lifestyle choices (drinking and smoking) and underlying illnesses (hypertension, diabetes, coronary heart disease, and hyperlipidemia) were gathered. The individuals' cognitive performance was evaluated using the Montreal Cognitive Scale (MoCA), and their serum NGAL levels were discovered using ELISA.

**Results:**

The investigation included 72 controls and 100 SCI patients. The baseline data did not differ substantially between the two groups, however the SCI group's serum NGAL level was higher than the control group's (*p* < 0.05), and this elevated level was adversely connected with the MoCA score (*p* < 0.05). According to the results of the ROC analysis, NGAL had a sensitivity of 58.24% and a specificity of 86.72% for predicting cognitive impairment following SCI.

**Conclusions:**

The changes in serum NGAL level could serve as a biomarker for cognitive impairment in SCI patients, and this holds true even after taking in account several confounding variables.

## 1. Introduction

Spinal cord injury (SCI) can occur at any level of the spinal cord and can result in temporary or permanent functional changes (1, 2). Symptoms and prognosis vary depending on the location and severity of the injury (3, 4). In most cases, the injury comes from physical trauma, and more than half of all injuries affect the cervical spine (5). In the US, there are roughly 20,000 new instances of SCI each year, and the per-person lifetime economic cost can be as high as 3 million US dollars (6, 7). The annual socioeconomic impact of SCI is projected to be 2.67 billion US dollars (8). Therefore, early identification of potential biomarkers that can effectively predict disease severity and prognosis in SCI may be the key to treating cognitive decline after SCI.

Neutrophil gelatinase-associated lipocalcin (NGAL), also known as lipocalcin-2 (LCN2) or oncogene 24p3 or, is an immune protein encoded by the LCN2 gene with a molecular weight of 25 kDa, and its immunomodulatory mechanism may be by limiting the utilization of iron by bacteria, it limits bacterial growth (9–11). *In vivo*, NGAL is mainly expressed in neutrophils, which is generally regarded as a biomarker of renal impairment (12, 13). In 1989, NGAL was first isolated from SV-40-infected mouse kidney cells by Hraba-Renevey et al. (14). Human NGAL contains a 20 amino acid signal peptide and a “lipidin” domain at the N-terminus of the protein, which exerts biological effects by binding to corresponding ligands (15). Human NGAL is up to 98% homologous to chimpanzees, but 62% and 63% homologous to mice and rats, respectively (15, 16).

The role of NGAL in neuroplasticity and its effects on cognitive function have been documented in recent years, but its precise mechanism is still poorly understood (17). Our purpose of this study is to further verify whether NGAL is involved in cognitive decline after SCI, in order to provide new biomarker targets for the prevention and treatment of cognitive impairment after SCI.

## 2. Methods

### 2.1. Study population

**Figure 1:**
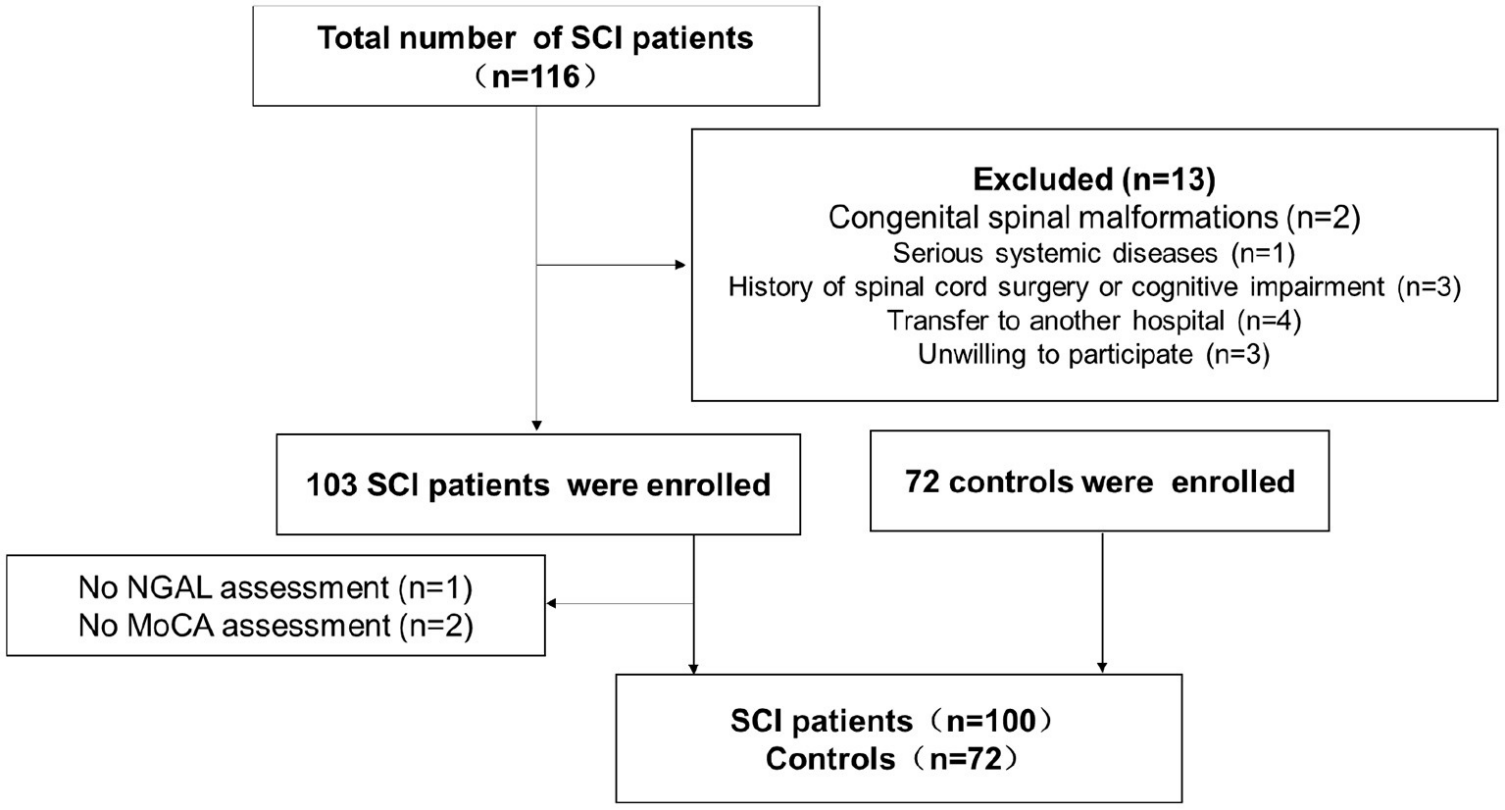
The flow chart of study implementation.

Patients with SCI and healthy controls who were hospitalized for Neck-Shoulder and Lumbocrural Pain Hospital between September 2020 and August 2022 made up the study population. The most recent recommendations provide the basis for SCI diagnosis. Congenital spinal abnormalities, severe systemic disorders, a history of spinal cord surgery, cognitive impairment, transfer to another hospital, and unwillingness to cooperate are the exclusion criteria for SCI. Additionally, a control group was drawn from the general population. All participants signed informed consent, and our study was approved by the hospital ethics committee (No. 2022012). The detailed flowchart is shown in Figure 1.

### 2.2. Baseline data

We collected baseline data including age, gender, education level, living habits (smoking and drinking), and underlying diseases (hypertension, diabetes, coronary heart disease, and hyperlipidemia). These data are obtained through questionnaires and recorded, counted and analyzed by specialized personnel.

### 2.3. Cognitive function

In this study, the MoCA scale, a popular measure for assessing cognitive function, was utilized to identify the cognitive function of SCI patients. The MoCA scale, developed by Montrealer Ziad Nasreddine, has been used by researchers and physicians worldwide since it was first released in 1996. It has been translated into 46 languages. For doctors, free. People were given 10 min to respond to 30 questions; the total score was 30, with one point deducted for each incorrect response, and a score of < 26 being judged cognitively deficient (18). Our study was approved by MoCA Test Inc. The evaluators were specially trained and blinded to the baseline data of the test subjects.

### 2.4. Serum NGAL level

Venous blood was collected immediately after fasting for 8 h after enrollment in all participants. Venous blood was allowed to stand at room temperature for 10 min, then centrifuged at 1200 g for 15 min, and the upper serum was separated and aliquoted and stored at −80°C for future use (19). ThermoFisher, Wilmington, DE, USA, provided the ELISA kit that was utilized to measure the presence of NGAL in the serum of SCI patients.

### 2.5. Statistical analysis

The statistical analysis was performed using SPSS 26.0. The measurement data was represented by the mean ± standard deviation (SD), while the enumeration data was represented by number (N). The link between serum NGAL and MoCA was discovered using P for trend. ROC analysis further evaluated the sensitivity and specificity of serum NGAL in predicting cognitive function in SCI. All statistical cutoffs were set at 0.05 and *p* < 0.05 was considered statistically significant.

## 3. Results

### 3.1. Clinical baseline data of the study population

We recruited a total of 100 SCI patients and 72 healthy controls for this study. The baseline demographic and clinical information for the complete study population, stratified by clinical traits, is shown in Table 1. Age, sex, education level, smoking, drinking, hypertension, coronary heart disease, diabetes, and hyperlipidemia were not statistically significantly different between the two groups, as shown in the table (*p* > 0.05).

**Table 1:** Demographic and clinical characteristics from the study population.

	**Controls (*n =* 72)**	**SCI (*n =* 100)**	***p*-value**
Age, years	59.3 ± 7.1	60.6 ± 7.8	0.265
Gender, male/female	56/16	88/12	0.073
**Education level**, ***n*** **(%)**			0.798
Low	33	51	
Middle	26	33	
High	13	16	
**Smoking**, ***n*** **(%)**			0.520
Never	26	38	
Former	10	17	
Current	36	45	
Drinking, *n* (%)	28	42	0.682
Hypertension, *n* (%)	20	32	0.552
Diabetes, *n* (%)	11	14	0.815
Coronary heart disease, *n* (%)	9	13	0.923
Hyperlipidemia, *n* (%)	17	28	0.518
NGAL, pg/ml	125.4 ± 12.3	196.7 ± 23.8	< 0.001
MoCA, points	27.5 ± 1.2	24.6 ± 1.7	< 0.001

NGAL, Neutrophil gelatinase-associated lipocalin; MoCA, Montreal cognitive test.

### 3.2. Serum NGAL level and MoCA score

**Figure 2:**
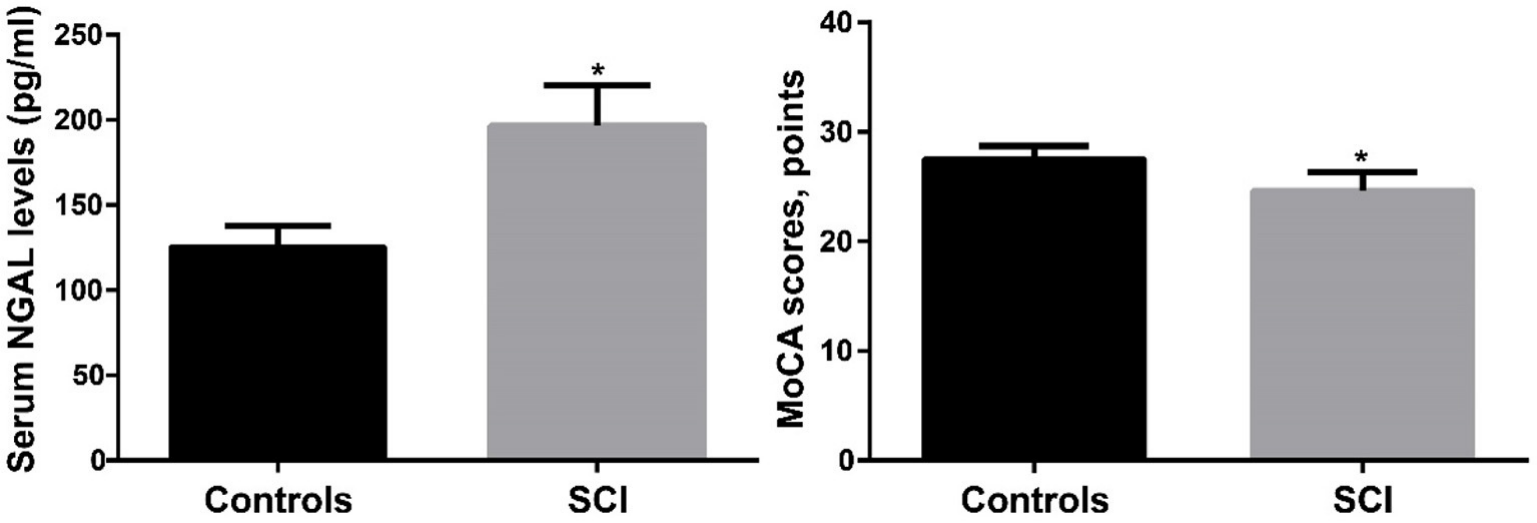
The comparison of serum NGAL levels and MoCA scores between SCI and controls. SCI, spinal cord injury. *p < 0.05.

The serum NGAL level for the control group was (125.4 ± 12.3 pg/ml, as reported in Table 1, while it was (196.72 ± 3.8 pg/ml) for the SCI group. The SCI group's serum NGAL levels were noticeably greater than those of the control group (*p* < 0.001). The SCI group's MoCA score was (24.6 ± 1.7) points, compared to the control group's (27.5 ± 1.2) points. When compared to the control group, the MoCA score of the SCI group was considerably lower (*p* < 0.001). Figure 2 compares the MoCA ratings and serum NGAL concentrations between the two groups. The results showed that the serum NGAL level in SCI group was significantly higher than that in control group, while the MoCA score was significantly lower than that in control group.

### 3.3. Correlation analysis between serum NGAL level and MoCA assessment

We separated the SCI patients into 4 groups based on the quartile levels of the serum NGAL and looked at the connection between those groups and the MoCA score. Table 2 displays the correlation analysis between the serum NGAL level and MoCA score. From Q1 to Q4, MoCA scores were (25.8 ± 1.9), (24.9 ± 1.8), (24.2 ± 1.5) and (23.5 ± 1.6), respectively. The findings indicated that the MoCA score decreased as blood NGAL level increased (*p* < 0.001), indicating that a high serum NGAL level may be a sign of cognitive impairment.

**Table 2:** Correlation analysis between serum NGAL levels and MoCA scores.

**Variable**	**Q1**	**Q2**	**Q3**	**Q4**	***P*-values**
MoCA scores	25.8 ± 1.9	24.9 ± 1.8	24.2 ± 1.5	23.5 ± 1.6	< 0.001

NGAL, Neutrophil gelatinase-associated lipocalin; MoCA, Montreal cognitive test.

### 3.4. Multiple model regression analysis

To explore the etiology affecting the MoCA score, we performed a multi-model regression analysis (Table 3). In model 1, after adjusting the confounding of age, sex and education level, it was suggested that serum NGAL was a risk factor for SCI-related cognitive impairment (*p* < 0.05); in model 2, we further adjusted the Smoking and drinking suggest that serum NGAL is also a risk factor for SCI-related cognitive impairment (*p* < 0.05); Serum NGAL was found to be an independent risk factor for cognitive impairment caused by SCI in model 3, which was based on model 2. After further adjusting for the underlying conditions (hypertension, diabetes, coronary heart disease, and hyperlipidemia), the same result was found (*p* = 0.047).

**Table 3:** Regression analysis of serum NGAL levels and MoCA scores.

	**MoCA scores**	
	**Regression coefficient**	***P*** **-values**
Model 1	0.352	< 0.001
Model 2	0.271	< 0.001
Model 3	0.218	0.047

Model 1: adjusted for age, gender and education levels; Model 2: further adjusted for smoking and drinking; Model 3: further adjusted for Hypertension, Diabetes, Coronary heart disease and Hyperlipidemia. NGAL, Neutrophil gelatinase-associated lipocalin; MoCA, Montreal cognitive test.

### 3.5. ROC curve analysis

**Figure 3:**
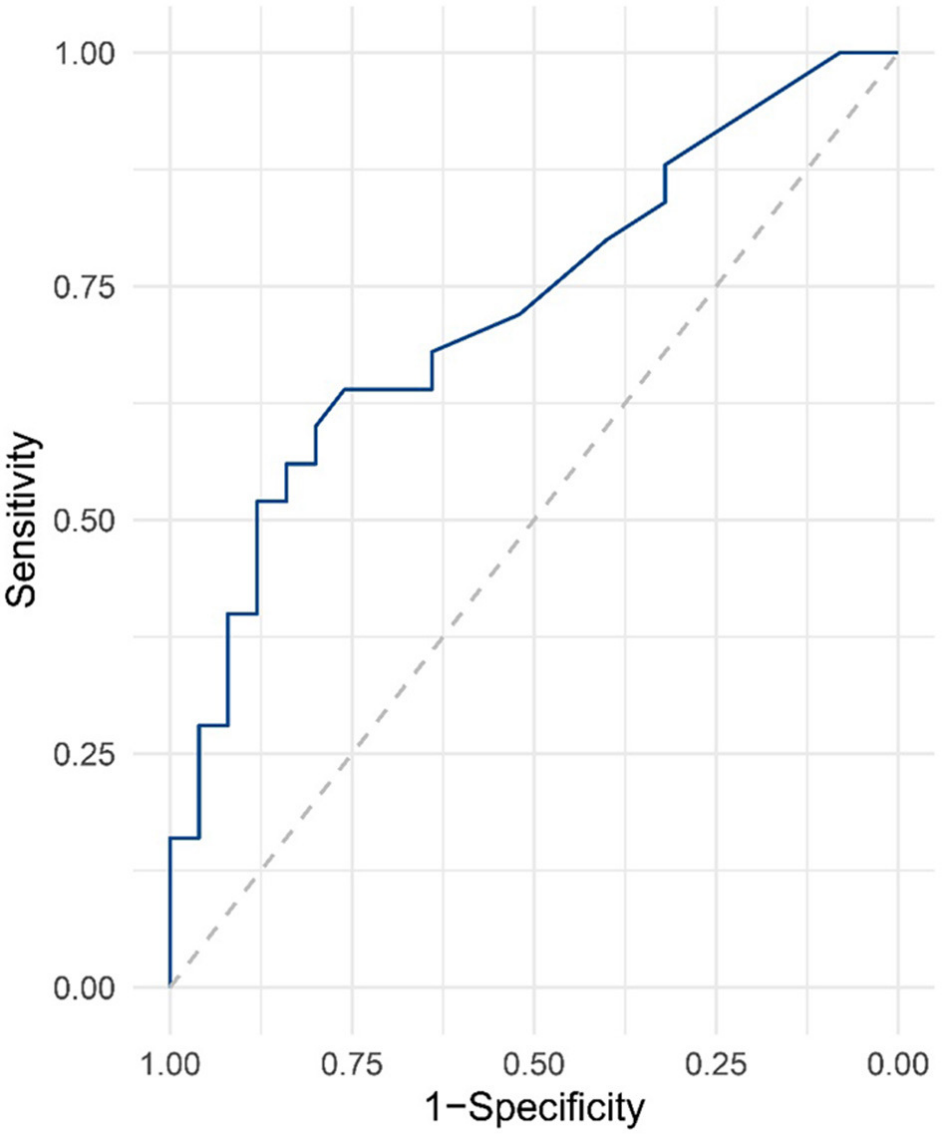
ROC curve analysis for serum NGAL levels in SCI.

In order to further verify the accuracy of serum NGAL level in diagnosing cognitive impairment after SCI, we performed ROC curve analysis, and the results are shown in Figure 3. The sensitivity of serum NGAL in diagnosing cognitive impairment after SCI was 72.48%, and the specificity was 61.28%.

## 4. Discussion

This is the first study of the relationship between cognitive impairment and serum NGAL levels in patients after SCI. Our results showed that the blood level of NGAL in SCI patients was significantly higher than that in healthy controls, and the quartile level was negatively correlated with MoCA score. In multi-model regression analysis, serum NGAL levels were considered to be independent risk factors for SCI-related cognitive impairment after adjusting for multiple confounding. Subsequent ROC analysis further proved that the serum NGAL level has a high accuracy in diagnosing SCI-related cognitive impairment. Our study suggests that serum NGAL levels may serve as a potential biomarker of SCI-related cognitive function.

NGAL is mainly expressed and secreted by immune cells, liver cells or renal tubular cells, and it can capture and consume siderophore to play an antibacterial role (20, 21). In addition to antibacterial, NGAL can also be used as a factor regulating cell growth and differentiation, mediating the biological activity of iron inside and outside cells (22). Genomic studies have shown that NGAL is one of the most up-regulated genes in acute kidney injury, and it can regulate the secretion of a renal tubulin with a molecular weight of 25KDa, which quickly enters the body fluid after the onset of renal injury (23). NGAL rises 24–48 h earlier than conventional serum creatinine, making it potentially a more effective biomarker. Studies have also shown that elevated levels of NGAL can predict the prognosis of acute kidney injury (24). All of the above make NGAL the focus of clinical translational research.

In addition to its involvement in acute kidney injury, a role for NGAL in neurological disorders has also been found. Zhao et al. (25) found that the expression of NGAL increased after traumatic brain injury, which was negatively correlated with the clinical score reflecting the severity of traumatic brain injury, and it has good sensitivity and specificity as a biomarker for diagnosing traumatic brain injury (25). Peng et al. (26) found that the level of NGAL increases after cerebral ischemia, and the activation of EGF/EGFR can regulate the expression of NGAL by activating the JAK2/STAT3 pathway to improve neurological deficits (26). Serra et al. (27) discovered that plasma NGAL levels were significantly higher in aneurysm patients than in the control group, indicating that NGAL may be involved in the pathophysiological process of aneurysms and that NGAL may be used as an indicator for assessing aneurysm rupture and prognosis in the future (27).

In recent years, studies on the involvement of NGAL in cognitive impairment have been found. The Dutch research team found that low levels of NGAL in serum and cerebrospinal fluid can be used as potential biomarkers to predict the conversion of mild cognitive impairment to Alzheimer's disease (AD), and affect the pathophysiological process of AD accompanied by depression (28, 29). The same research team also found that NGAL was associated with cognitive impairment in patients with depression, and there were gender differences (30). In addition, NGAL is also considered to be associated with the pathogenesis of Down syndrome.

The research of NGAL in SCI has also come into the field of vision of researchers. Behrens, V found that NGAL was significantly increased in the spinal cord, brain, liver and serum in the SCI mouse model, while the absence of NGAL could significantly reduce the differentiation of glial cells, indicating that it may be involved in the inflammatory injury after SCI (31). Rathore et al. (32) found that Lcn2 can regulate the inflammatory response after SCI, while the lack of NGAL can reduce the secondary injury after SCI and improve the recovery of motor function (32). However, clinical studies of NGAL in SCI patients have not been reported.

The first study to reveal cognitive damage in patients following NGAL involvement in SCI is ours. However, our study has certain flaws. Our study is a single-center, small-sample investigation with Chinese participants. It is debatable if the findings of this study apply to other geographic or racial groups. There is an urgent need for large-sample multi-center research to confirm this study.

## 5. Conclusions

The results of the current study suggest that changes in serum NGAL could serve as a biomarker for cognitive impairment in SCI patients, and this finding holds true even after taking into account a number of confounding variables. To develop innovative methods for treating cognitive impairment caused by SCI, future scientific and clinical research must further investigate the underlying mechanism.

## Data availability statement

The raw data supporting the conclusions of this article will be made available by the authors, without undue reservation.

## Ethics statement

The studies involving human participants were reviewed and approved by the Ethics Committee of Neck-Shoulder and Lumbocrural Pain Hospital. The patients/participants provided their written informed consent to participate in this study.

## Author contributions

QZ and YC designed this research and wrote the manuscript. ZL, LX, SC, ZC, and BS participated in data collection, experimental process, and data analysis. All authors contributed to the article and approved the submitted version.
